# Differential changes in the adenoids and tonsils in Japanese children and teenagers: a cross-sectional study

**DOI:** 10.1038/s41598-017-09893-9

**Published:** 2017-08-29

**Authors:** Asuka Manabe, Takayoshi Ishida, Hyung Sik Yoon, Shin-Sheng Yang, Eiichiro Kanda, Takashi Ono

**Affiliations:** 1 Department of Orthodontic Sciences, Tokyo Medical Dental University Graduate School, Tokyo, Japan; 2 All Barun Dental Clinic, Suwon, Korea; 3 Department of Nephrology and Hypertension, Tokyo Kyosai Hospital, Tokyo, Japan

## Abstract

When adenoids (Ad) and tonsils (Tn) become hypertrophied, craniofacial and general body growth is affected. However, there are no objective explicit criteria for evaluating the size of the Ad and Tn, and their respective growth patterns remain unclear. This study determined the average proportions of the Ad and Tn sizes to the upper airway area at various developmental stages in Japanese individuals. Lateral cephalometric radiographs were obtained from 300 Japanese children and teenagers (150 boys and 150 girls, aged 6–20 years), and the respective proportions of Ad to the nasopharynx (Ad/Np) and Tn to the oropharynx (Tn/Op) in the upper airway were determined. Average and standard deviation (SD) were calculated for each of the 5 age groups: lower primary school, upper primary school, junior high school, senior high school, and young adults. We investigated the correlation between age and growth patterns of Ad and Tn, and determined the average Ad/Np and Tn/Op. There was an age-related decrease in Ad and Tn size, and a significant positive correlation between Ad/Np and Tn/Op values in the upper primary school group. Both Ad/Np and Tn/Op decrease as individuals approach adulthood. However, the growth patterns of the Ad/Np and Tn/Op differ from each other.

## Introduction

Both the adenoids (Ad) and tonsils (Tn) are located at the entrance of the upper airway and are immunological tissues that are stimulated by foreign antigens passing through this region^1^. Immunity increases with growth and development^2, 3^; Scammon’s curves of systemic growth indicate the change in the size of organs with growth and the development of the postnatal individual to adulthood^4^. Among the 4 types of growth curves, lymphoid tissue, such as the Ad, shows a unique growth pattern, including overgrowth and involution. Organs belonging to the lymphoid type attain approximately 200% growth by late childhood, and then undergo involution by adulthood. In addition to the thymus and the spleen also belong to the lymphoid type^4^. However, the mechanism of overgrowth and subsequent involution of the Ad and Tn have not been clearly elucidated^5^.

In a selected number of individuals, the Ad and Tn might remain overgrown, without undergoing involution. The overgrown Ad and Tn can induce a developmental disorder in the craniofacial region by obstructing the upper airway and changing respiratory conditions^6, 7^. It has also been reported that adeno-tonsillar hypertrophy can affect growth in height, weight gain, and cardiopulmonary function^8, 9^. Recently, it has been shown that adeno-tonsillar hypertrophy is the most common cause of obstructive sleep apnoea (OSA) in children; similarly adeno-tonsillar hypertrophy in adults is one of the reasons of sleep-disordered breathing (SDB), featuring obstructive hypoventilation, snoring, and upper airway narrowing^8^. It has been revealed that a qualitative evaluation of adeno-tonsillar hypertrophy is insufficient for evaluation of the severity of SDB^6, 10^. However, no reports have assessed the size of the Ad and Tn relative to the upper airway in the same individual. Therefore, there is a need to develop a credible method for evaluating the size of the Ad and Tn.

Hence, the purpose of this study was (1) to determine the average proportion of the size of the Ad and Tn to the area of the upper airway, (2) to compare the growth pattern of the Ad and Tn, at various stages of development, with reference to the interaction between the Ad and Tn and systemic growth, (3) with a view to determining whether adeno-tonsillectomy should be performed.

## Results

Three hundred subjects were enrolled in this study. The sample was subdivided into 5 groups on the basis of age: lower primary school [age: 8.1 ± 0.7 (mean ± standard deviation) years, n = 51], upper primary school (10.3 ± 0.8 years, n = 105), junior high school (13.6 ± 0.9 years, n = 37), senior high school (16.4 ± 0.8 years, n = 42), and young adults (19.3 ± 0.8 years, n = 65). The composition of each group is shown in Table 1. There was no significant difference in the sex ratio in each group.

**Table 1:** Study population. There was no significant difference in the ratio of males to females in each group.

	Lower primary school	Upper primary school	Junior high school	Senior high school	Young Adults	*p* value
N	51	105	37	42	65	
Age	8.7 ± 0.7	10.3 ± 0.8	13.6 ± 0.9	16.4 ± 0.8	19.3 ± 0.8	0.000*
Sex, male (%)	26 (51.0%)	50 (47.6%)	19 (51.4%)	19 (45.2%)	36 (55.4%)	0.842

*p < 0.05.

**Figure 1:**
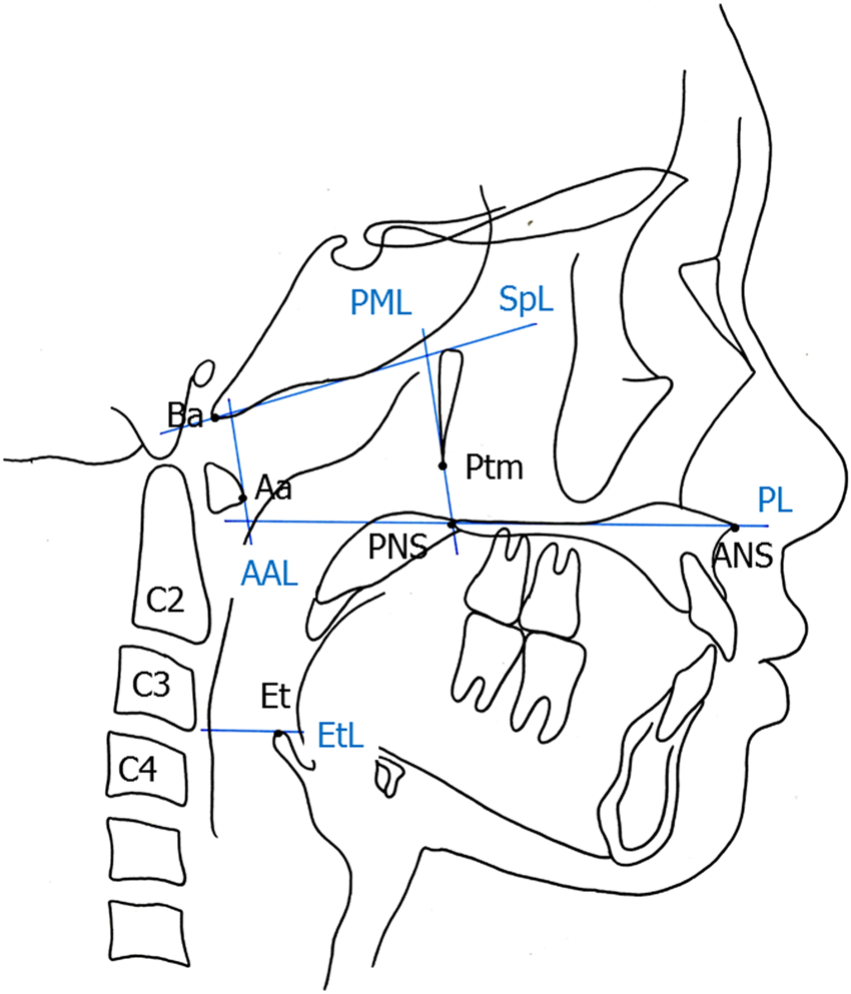
Landmarks and reference planes of the nasopharynx, oropharynx, and hypopharynx. Abbreviations: ***Aa***, anterior medial point of the atlas; ***ANS***, anterior nasal spine; ***Ba***, Basion; ***C2***, second cervical vertebra; ***C3***, third cervical vertebra; ***C4***, fourth cervical vertebra; ***Et***, epiglottis; ***PNS***, posterior nasal spine; ***Ptm***, pterygomaxillary fissure; ***AAL***, anterior atlas line (a line parallel line to the pterygomaxillary line registered on the most front point of the atlas); ***SpL***, sphenoid line (a line tangential to the lower border of the sphenoid bone registered on the basion); ***EtL***, epiglottis line (a line parallel to the palatal line registered on the most superior point of the epiglottis); ***PL***, palatal line (a line from the anterior nasal spine to the posterior nasal spine); ***PML***, pterygomaxillary line (a line perpendicular to the palatal line registered on the pterygomaxillon).

**Figure 2:**
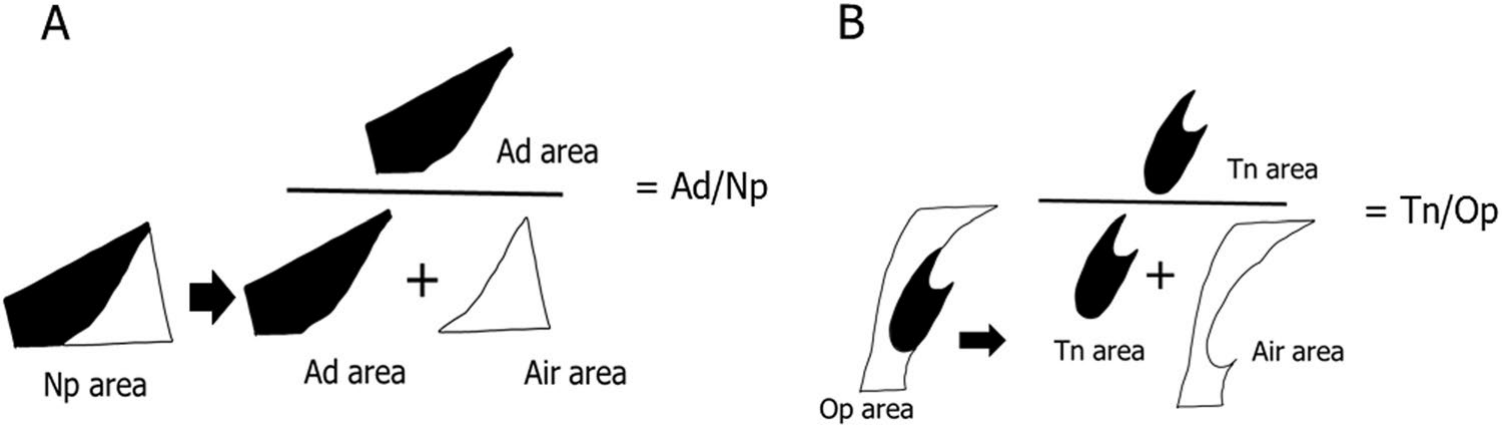
Definitions of the adenoidal area to the nasopharyngeal area ratio (**A**) and the tonsillar area to the oropharyngeal area ratio (**B**). Abbreviations: ***Ad area***, a cross-sectional area of the adenoidal (Ad) tissue; ***Np area***, a cross-sectional area of the nasopharynx; ***Air area***, a cross-sectional area of the upper airway; ***Ad***/***Np***, Ad area/Np area ratio; ***Op area***, a cross-sectional area of the oropharynx; ***Tn area***, a cross-sectional area of the tonsillar (Tn) tissues; ***Tn***/***Op***, Tn area/Op area ratio.

**Figure 3:**
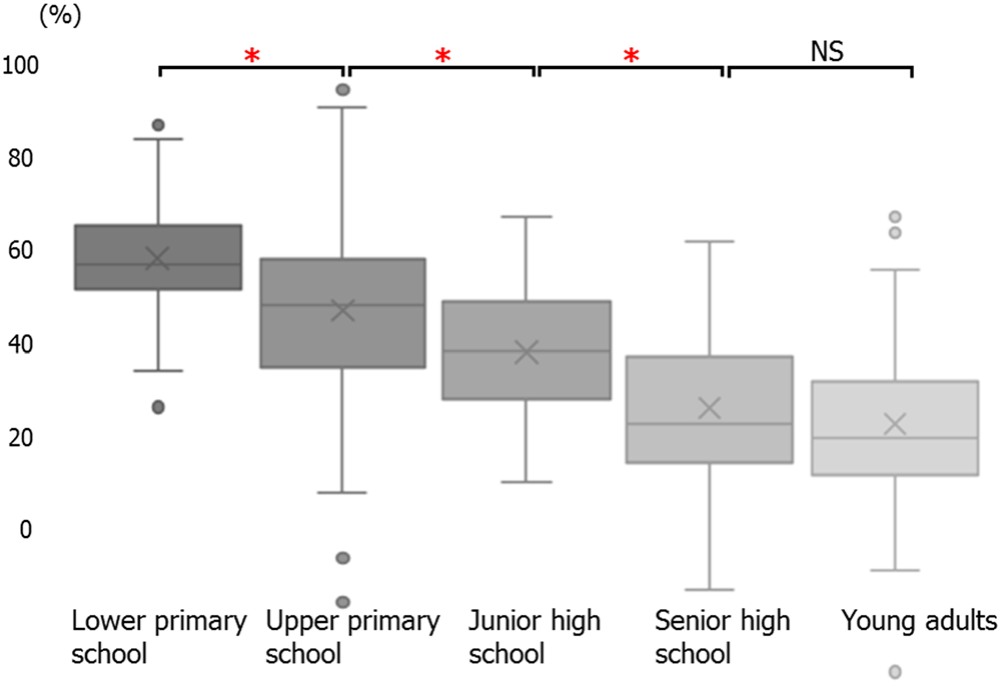
Age-dependent change in the Ad/Np. The values for the Ad/Np were 60.7 ± 7.8 for the lower primary school group, 53.2 ± 12.5 for the upper primary school group, 47.2 ± 9.2 for the junior high school group, 39.1 ± 11.9 for the senior high school group, and 36.9 ± 12.1 for the young adults group. The notches on the box plots indicate the median. The box indicates the inter-quartile range (IQR), being the difference between the third and first quartiles. The cross indicates the mean. Up and down plots are outliers. *p < 0.0125.

**Figure 4:**
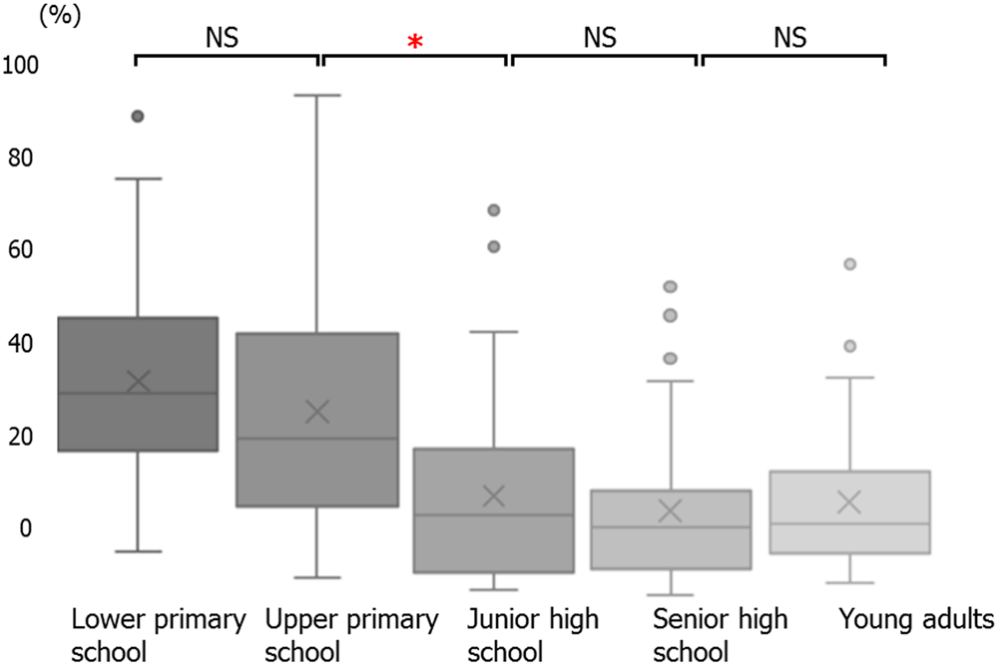
Age-dependent change in the Tn/Op. The values for the Tn/Op were 32.5 ± 15.1 for the lower primary school group, 28.0 ± 16.9 for the upper primary school group, 15.5 ± 13.4 for the junior high school group, 13.4 ± 10.8 for the senior high school group, and 10.8 ± 14.6 for the young adults group. The notches on the box plots indicate the median. The box indicates the interquartile range (IQR), being the difference between the third and first quartiles. The cross indicates the mean. Up and down plots are outliers. *p < 0.0125.

We calculated the ratio of the cross-sectional area of the Ad and the Tn to that of the pharyngeal airway on pre-treatment lateral cephalometric radiographs. These radiographs were obtained under standardized conditions. We divided the area into 4 parts following the methods described in a previous study (Fig. 1)^6^. The proportion of the nasopharyngeal area (Np) taken up by the Ad (Ad/Np) was calculated by dividing the adenoidal area by the trapezoidal area made up by the palatal line, sphenoid line, anterior atlas line, and pterygomaxillary line (Fig. 2A). The proportion of the oropharyngeal area (Op) taken up by the Tn (Tn/Op) was calculated by dividing the tonsillar area by the area outlined by the inferior border of the nasopharynx, the posterior surface of the soft palate, the postero-inferior surface of the tongue, the epiglottis line and the posterior pharyngeal wall (Fig. 2B). The average value of Ad/Np was 60.71 ± 7.81 mm^2^ in the lower primary school, 53.23 ± 12.49 mm^2^ in the upper primary school, 47.18 ± 9.19 mm^2^ in the junior high school, 39.08 ± 11.88 mm^2^ in the senior high school, and 36.86 ± 12.12 mm^2^ in the adult groups. There was a significant decrease in the Ad/Np value in the junior high school group as compared to the lower primary school group and in the lower primary school group as compared to the junior high school group (Fig. 3). On the other hand, the average values of Tn/Op were 32.51 ± 15.12 mm^2^ in the lower primary school, 27.95 ± 16.93 mm^2^ in upper primary school, 15.53 ± 13.36 mm^2^ in the junior high school, 13.41 ± 10.78 mm^2^ in the senior high school, and 14.61 ± 10.10 mm^2^ in the adult groups. There was a significant decrease in the Tn/OP value in the junior high school group as compared to the lower and upper primary school groups (Fig. 4). A significant decrease in Ad/Np and Tn/Op were observed from the lower primary school groups to the young adult groups. There was thus an age-related decrease in Ad/Np and Tn/Op in the age range studied here. Ad/Np and Tn/Op have independent relationships after adjusting for sex and age group, converted to dummy variables (β = 0.145, *p* = 0.002). There was also an independent relationship between Ad/Np and sex (β = −3.007, *p* = 0.020).

When multiple regression analysis was performed for each age group, Ad/Np and Tn/Op had independent relationships in the upper primary group. No correlation was found in other groups (Table 2).

**Table 2:** Results of multivariable logistic regression analysis of the associations between Ad/Np and Tn/Op.

	β	SE	*P* value
Lower primary school	0.122	0.072	0.099
Upper primary school	0.205	0.070	0.004 *
Junior high school	0.00525	0.117	0.965
Senior high school	0.243	0.165	0.148
Young Adults	0.00226	0.148	0.988

Ad/Np = β (Tn/Op) + β (sex). *p < 0.05.

## Discussion

Several studies have reported that enlarged Ad and Tn influence the general growth and development of the body^11, 12^. However, there is limited information available about age-dependent growth and development of the Ad and Tn. The most important contributing factor of OSA, especially during childhood, is upper respiratory obstruction caused by adeno-tonsillar hypertrophy^13^.

A recent study reported that anatomical abnormalities in the upper airway can structurally obstruct the normal airway and can lead to abnormal airway occupancy^14^. In this study, we investigated the growth and development of the Ad and Tn across various ages, using an indication of airway occupancy that reveals the influence on respiration. We used cephalometric radiographs to evaluate the influence on respiration objectively by observing the airway occupancy of the Ad and Tn across different age groups. This may be helpful in the differential diagnosis of adeno-tonsillar hypertrophy.

A significant correlation between the Ad/Np and Tn/Op at cervical vertebral maturation stage (CVMS) 2–3, which corresponds to the upper primary school age^15^, has previously been reported^6^. Therefore, our findings were in line with that of the previous study. It has also been reported that the antero-posterior diameter of the Np increased in the upper primary school age and junior high school age children, and that that of the Op increased in the lower primary school age and junior high school age children, indicating that the Np and Op undergo growth spurts with different timings^16, 17^. The embryological origins of the Ad and Tn differ; the Ad develops from the third pharyngeal arch, while the Tn develops from the second pharyngeal arch^1, 18^. Moreover, the Ad and Tn show different histological structures in that there are differences in the epithelium and capsule. Both the decrease in the Ad/Np and Tn/Op indicated that the atrophy of the Ad and Tn themselves and the enlargement of the Np and Op could be involved. It is thought that differences between the growth pattern of the Np and Op as denominators and the growth pattern of the Ad and Tn as the respective numerators are factors in the difference in development pattern of the airway occupancy ratio. Furthermore, the age at which the decrease in the Ad/Np occurs, in children in the lower primary school age, where the denominator does not decrease, is considered to be the time during which the Ad itself regresses. However, a longitudinal investigation in the same individuals is necessary to elucidate the growth and development of the Ad and Tn, given the cross-sectional nature of the present survey.

Adeno-tonsillectomy is the most common treatment option for adeno-tonsillar hypertrophy and is also the major surgery performed in otolaryngology. According to the guidelines of the American Academy of Paediatrics^19^, adeno-tonsillectomy is the first-line treatment for childhood OSA syndrome^20^. Adeno-tonsillar hypertrophy has typically been diagnosed by qualitative standards. However, the adenoidal-nasopharyngeal ratio (ANR), which is a quantitative measurement method using lateral cephalometric radiographs for evaluating the ratio occupied by the Ad, was advocated by Fujioka and co-workers^21^. The ANR is obtained by dividing the adenoidal depth (i.e., the distance between the maximum convexity of the Ad shadow and the tangent line of the front edge of the sphenoid bone) by the nasopharyngeal depth (i.e., the distance between the posterior superior edge of the hard palate and the antero-inferior edge of the spheno-basi-occipital synchondrosis)^21^. To the best of our knowledge, there have been no previous reports describing the development of the Ad and Tn at the same time, other than our previous study. In that study, we showed a correlation between the airway occupancy of the Ad and Tn at the certain developmental stages^6^.

It has been reported that evaluation by means of X-ray, based on the ANR, shows a significant correlation with endoscopic evaluation^21, 22^. X-rays also have the advantage that they can be obtained even in young patients, in whom endoscopy may not be applicable. In our study, we performed a quantitative analysis of the proportion of the upper airway taken up by the Ad and Tn, which can further increase the effectiveness of the X-ray evaluations.

In this study, we used the cephalometric radiograph taken before orthodontic treatment from the subjects aged 6–20 years. Cephalometry is a method for standardizing the cephalometric head film, introduced by Broadbent in 1931^23^, and to date has been commonly used as one of the routine examinations in orthodontic diagnoses, prior to starting orthodontic treatment. Cephalometric radiographs have been shown to yield highly reproducible data in many orthodontic studies^24–26^. Since both the source-to-subject and subject-to-film distances are always fixed, it is possible to calculate the magnification of the subject projected on the film in a precise manner^27–29^. It is also possible to correct the actual measured value with radiographic image magnification. Therefore, by using the cephalometric radiograph, it is possible to evaluate growth and development over time, and it is therefore considered an effective tool in longitudinal studies.

There are several limitations to our study. First, we used 2-dimensional lateral cephalometric radiography. However, standardized lateral cephalometric radiographs are reproducible and there is no concern about additional radiological exposure. In fact, lateral cephalometric radiographs are essential resources that are routinely used for orthodontic diagnoses. Evaluation of the upper airway area by analysis of 2-dimensional lateral cephalometric radiographs is highly correlated with 3-dimensional upper airway assessment and can be used as a screening test for predicting airway volume prior to using computed tomography^30^. Second, the present study was a cross-sectional study, where the individuals’ growth was not followed-up over time. There is therefore a need for a longitudinal study to follow the growth of the Ad and Tn in the same individuals.

## Conclusions

In the present study, there was a significant decrease in the size of the Ad and Tn relative to the upper airway, from the ages of 6 to 20 years, and we presented the average and standard deviation of these proportional areas according to age. Both the Ad/Np and Tn/Op decrease as individuals approach the adulthood. However, the growth patterns of the Ad/Np and Tn/Op differ from each other.

## Materials and Methods

Permission for this cross-sectional clinical study was obtained from the Research Ethics Committee (Permission number: D2015-626) of Tokyo Medical and Dental University Dental Hospital (Tokyo, Japan). Informed consent was obtained from either the study patients or the patients’ parents. Additionally, all of the experiments were performed in accordance with the relevant guidelines and regulations. The study involved 300 Japanese children (150 boys and 150 girls; age: 6–20 years) randomly selected from patients who visited Tokyo Medical and Dental University Dental Hospital. No subjects had undergone adenoidal or tonsillar surgery. The a-priori sample size estimation was performed at the 5% level of significance (α = 0.05), with a power of 80%, and revealed that a minimum of 26 subjects was necessary per age group.

All cephalometric radiographs were taken according to the internationally popular settings^27–29, 31^. When taking a cephalometric radiograph, the patient’s head was fixed with ear rods, and the Frankfurt plane was set to be parallel to the floor. The source-to-subject and subject-to-film distances were always fixed^31^. The Ad/Np was calculated by dividing the adenoidal area by the trapezoidal area made up by the palatal line, sphenoid line, anterior atlas line, and pterygomaxillary line (Fig. 2A). The Tn/Op was calculated by dividing the tonsillar area by the area outlined by the inferior border of the nasopharynx, the posterior surface of the soft palate, the postero-inferior surface of the tongue, the epiglottis line and the posterior pharyngeal wall (Fig. 2B). Each of the 4 areas were measured 3 times on 3 different days, using Winceph ver.9.0 software (Rise Corp., Tokyo, Japan), and the average value of each area were used. The lateral cephalometric radiographs were traced and analysed by a single investigator (A.M.).

Statistical analyses were performed to determine possible correlations among groups. All areas were randomly re-measured and errors were calculated by Dahlberg’s formula^24^; on average, the method error was 1.4 mm^2^ (1.4 mm^2^ for the Ad, 1.9 mm^2^ for the Np, 0.7 mm^2^ for the Tn, and 1.6 mm^2^ for the Op). Inter-group comparisons were carried out using one-way analysis of variance and chi-square test. Multiple comparisons were examined using Student’s *t*-test with the Bonferroni method (*p* < 0.0125). Next, multiple regression analysis was performed using Ad/Np as a dependent variable, Tn/Op, sex, and the age group converted to a dummy variable as independent variables. Furthermore, multiple regression analysis was performed for each age group, using Ad/Np as a dependent variable, and Tn/Op and sex as independent variables. Unless otherwise stated, *p* < 0.05 was regarded as indicating statistical significance. Statistical analyses were performed using SPSS (Statistical Package of Social Sciences, Chicago, IL, USA) software version 19.
